# Supplementary material for: The Systems Biology Research Tool: evolvable open-source software
Source: BMC Syst Biol. 2008 Jun 29;2:55. doi: 10.1186/1752-0509-2-55 (PMC2446383; doi:10.1186/1752-0509-2-55)
Supplement: Additional file 1 — SBRT Archive. An archive of the current version of the Systems Biology Research Tool. [file 1752-0509-2-55-S1.zip › sbrt-1.4.0/doc/developers_guide/api/sbrt/kernel/util/DiscreteProgressMonitor.html]

DiscreteProgressMonitor


|  |  |  |
| --- | --- | --- |
| **Overview** \| **Package** \| **Class** \| **Use** \| **Tree** \| **Deprecated** \| **Index** \| **Help** | |  |
| **PREV CLASS**   **NEXT CLASS** | **FRAMES**    **NO FRAMES**     **All Classes** |  |
| SUMMARY: NESTED \| FIELD \| CONSTR \| METHOD | DETAIL: FIELD \| CONSTR \| METHOD |  |


---


## sbrt.kernel.util Class DiscreteProgressMonitor

```
java.lang.Object
  sbrt.kernel.util.AbstractProgressMonitor
      sbrt.kernel.util.DiscreteProgressMonitor
```

**All Implemented Interfaces:**: MutableProgressMonitor, ProgressMonitor

---

``` public class DiscreteProgressMonitor extends AbstractProgressMonitor ```

This class is used to monitor the progress of a process that occurs in
discrete events.

**Author:**
:   This class was written and documented by
    Jeremiah Wright while in the Wagner lab.

---

**Constructor Summary**

`DiscreteProgressMonitor()`
  
          Constructs a new discrete progress monitor.


| **Method Summary** | |
| --- | --- |
| `ProgressMonitor` | `asUnmodifiable()`             Returns this progress monitor as an immutable object. |
| `java.lang.Integer` | `getProgress()`             Returns an integer value indicating a progress. |

**Methods inherited from class sbrt.kernel.util.AbstractProgressMonitor**

`getCount, getDuration, increment, resetCount, start, started, stop, stopped`

**Methods inherited from class java.lang.Object**

`clone, equals, finalize, getClass, hashCode, notify, notifyAll, toString, wait, wait, wait`

**Constructor Detail**

### DiscreteProgressMonitor

```
public DiscreteProgressMonitor()
```

:   Constructs a new discrete progress monitor.


**Method Detail**

### getProgress

```
public java.lang.Integer getProgress()
```

:   Returns an integer value indicating a progress.

    :   **Returns:**: an integer value indicating a progress.

---


### asUnmodifiable

```
public ProgressMonitor asUnmodifiable()
```

:   Returns this progress monitor as an immutable object. This allows
    the contents of this progress monitor to be queried without the
    possibility of modification.

    :   **Returns:**: this progress monitor as an immutable object.


---


|  |  |  |
| --- | --- | --- |
| **Overview** \| **Package** \| **Class** \| **Use** \| **Tree** \| **Deprecated** \| **Index** \| **Help** | |  |
| **PREV CLASS**   **NEXT CLASS** | **FRAMES**    **NO FRAMES**     **All Classes** |  |
| SUMMARY: NESTED \| FIELD \| CONSTR \| METHOD | DETAIL: FIELD \| CONSTR \| METHOD |  |


---
